# Sex differences in avoidance behavior after perceiving potential risk in mice

**DOI:** 10.1186/s12993-017-0126-3

**Published:** 2017-05-05

**Authors:** Sayaka Yokota, Yusuke Suzuki, Keigo Hamami, Akiko Harada, Shoji Komai

**Affiliations:** 1 Graduate School of Biological Sciences, Nara Institute of Science and Technology, 8916-5 Keihanna Science City, Nara, 630-0192 Japan; 2 Graduate School of Medicine, Kyoto University, Kyoto, Japan; 3 JST, PRESTO, Saitama, Japan

**Keywords:** Sex difference, Associative learning, Avoidance behavior, Mobility, Context, Behavioral test battery

## Abstract

**Background:**

Sex has been considered as a potential factor regulating individual behaviors in different contexts. Recently, findings on sex differences in the neuroendocrine circuit have expanded due to exact measurements and control of neuronal activity, while findings on sex differences in behavioral phenotypes are limited. One efficient way to determine the miscellaneous aspects of a sexually different behavior is to segment it into a set of simpler responses induced by discrete scenes.

**Methods:**

In the present study, we conducted a battery of behavioral tests within a variety of unique risky scenes, to determine where and how sex differences arise in responses under those scenes.

**Results:**

A significant sex difference was observed in the avoidance responses measured in the two-way active and the passive avoidance tests. The phenotype observed was higher mobility in male mice and reduced mobility in female mice, and required associative learning between an escapable risk and its predictive cue. This was limited in other scenes where escapable risk or predictive cue or both were missing.

**Conclusions:**

Taken together, the present study found that the primary sex difference occurs in mobility in the avoidance response after perceiving escapable risks.

**Electronic supplementary material:**

The online version of this article (doi:10.1186/s12993-017-0126-3) contains supplementary material, which is available to authorized users.

## Background

Sex has been considered as a potential variable regulating individual behavior in animals including humans under various contexts. Recently, sex differences in neuroendocrine circuits have been shown to exist even at a microscopic scale [1–5], owing to the development of techniques that allow for precise measurement and control of neuronal activity [1, 2, 6]. However, those phenotypes at a behavioral scale have been difficult to elucidate, since most behavior are composed of several responses closely associated with specific scenes [7–11]. To detect those unique responses, scene segmentation, i.e. to break down a general context into discrete behavioral tests would be a plausible approach. By identifying the responses and the conditions in which they occur, phenotypes of a behavior could be determined. In the present study, we conducted a test battery probing the responses to a variety of risky contexts, to determine where and how sex differences arise in these responses under those test conditions in mice.

In particular, we determined whether sex differences arise in the avoidance response, since it constitutes major components of a behavior under escapable risks [10, 12–18]. Avoidance can be further segmented into two sub-components, active and passive avoidance, depending on the cue that prompts it [11, 12, 19]. Active avoidance occurs when animals attempt to move away from a risk, whereas in passive avoidance, animals try to maintain a safe distance from such a threat [12, 20, 21], in response to cues predicting an escapable risk after associative learning has occurred. For the former sub-component, a two-way active avoidance test was carried out. In this test, we measured the formation of the association between a conditioned stimulus and an escapable unconditioned stimulus in a context, with subsequent active transfer during the tone allowing avoidance of the foot-shock simultaneously with the termination of the tone. For the latter, a passive avoidance test was used. In this test, we measured the formation of the association between a dark room and an inescapable foot-shock, and subsequent staying in the light compartment that constitutes the passive avoidance. Since associative learning is necessary to perceive a risk that are hidden in the environment [13], the formation of associative learning alone was also compared between sexes by using a fear-conditioning test including contextual fear conditioning and cued-fear conditioning. Three additional tests were prepared to confirm whether sex differences arise under the lack of escapable risks or risk prediction or both. In a light/light (L/L) test, risk-predicting cues were unavailable. For active and passive response to an aversive, inescapable scene, a forced-swim test and tail-suspension test were used. Further, sex differences in innate aversion were tested in a light/dark test (L/D), and the basal activity in a home cage was also compared between sexes.

## Methods

### Subjects

Subjects were sexually naïve, 7-week-old male and female C57BL/6N mice (Japan SLC, Shizuoka, Japan). Each animal was housed separately in a home cage in a standard laboratory environment, on a 12-h light/dark cycle, at a constant temperature (23–24 °C) and relative humidity (50–70%). Food (pellets; Japan SLC, Shizuoka, Japan) and water were available ad libitum. All behavioral tests were carried out in the light phase. We used different batches of mice for each behavioral test; the mice were randomly assigned to any one behavioral test when 8 weeks old, with 3–4 days of prior handling. Experiments were approved by the Animal Care Committee of Nara Institute of Science and Technology (the permit number: 1004) and conformed to all relevant regulatory standards.

### Determination of the estrous cycle phases

We compared the behaviors between males and females and also between females in different phases of the estrous cycle, as it is well known that both human and rodent females alter their behavior depending on the estrous cycle phase [21–24].

For all female mice, estrous cycle phase was determined by vaginal smear cytology analyses during the 4 days prior to handling. Briefly, we rinsed the vagina with 150–200 μL sterile water. The smear was placed on a glass slide (FRC-01, Matsunami Glass industries, Osaka, Japan). After drying, 50 μL of Giemsa stain solution (Merck, Tokyo, Japan) was applied to the smear, which was left to stand for 10–20 min and then washed with distilled water. After drying, the smear was observed under a light microscope (Nikon Diaphot 300, Nikon Corporation, Tokyo, Japan), and we then classified it as either proestrus, estrus, diestrus [25], or ‘not determined’ (nd) depending on the results of the analysis. To control for any behavioral effects of this procedure between sexes, males were also treated in the same way, with sterile water applied under the scrotum.

### Behavioral tests

#### Two-way active avoidance test

Initially, we examined whether any sex differences occurred in active avoidance. In this test, 14 male and 16 female (3 proestrus, 6 estrus, and 7 diestrus) mice were used. The mice were required to learn the association between an auditory cue and a nociceptive foot-shock stimulus, and to then avoid the foot-shock by perceiving the auditory cue, across trials. The experimental procedure was based on a previous study [26]. Briefly, mice were placed in 1 of 2 adjacent compartments, separated by a partition, in a shuttle box (height = 185 mm, width = 300 mm, depth = 115 mm; Passive Avoidance System, Bio-Medica, Osaka, Japan). Constant luminance was maintained in both compartments. Immediately after the partition was removed, the mice could move freely between the two compartments. One minute after placement, the auditory cue was presented for 5 s in the shock compartment. The final 2 s of cue presentation were accompanied by the foot-shock. This procedure was repeated for 100 trials. The inter-trial interval was set at 20 ± 3 s. The active avoidance rate was defined as the number of entries into the safe compartment across 100 trials.

To ensure that the foot-shock did not disrupt their behavior, the current intensity of foot-shock for males and females was set at their pain threshold, which was determined in a pilot study (males = 0.11 ± 0.005 mA, females = 0.09 ± 0.003 mA). The threshold was determined as the average of individual thresholds within the group, and these were measured as the minimum current that induced a jumping response when the intensity was gradually increased from 0.089 mA by manually adjusting the current controller.

#### Passive avoidance test

In the active avoidance test, animals faced a threatening context in which their mobility (the active movement between compartments) constituted an adaptive behavior to avoiding a threat. Next, we performed the passive avoidance test to test whether any sex differences existed in a converse situation where the subject’s immobility (staying in one compartment) would be more adaptive. Therefore, if a difference in the avoidance pattern between the sexes was observed in the active avoidance test, we predicted that the passive avoidance test would show the opposite result.

In the passive avoidance test, 11 male and 18 female (3 proestrus, 8 estrus, 6 diestrus, and 1 nd) mice were examined. While the experimental procedure was based on a previous study [27], the foot-shock current intensity was set at the pain threshold for both males and females similar to the active avoidance test (see above). The same shuttle box was used as in the active avoidance test, except that one compartment was darkened while normal illumination was maintained in the other.

This test comprised of training and test sessions. In the training session, mice learned the association between a dark compartment and foot-shock that would enable them to anticipate the upcoming foot-shock. The two compartments were initially separated by a partition. After a mouse was placed in the light compartment, the partition was removed. When the mouse entered the dark compartment, the partition was closed again. Ten seconds after entry, a foot-shock was applied for 2 s. Ten seconds later, the partition was opened and the mouse returned to the light compartment. Twenty-four hours after the training session, the test session was initiated. The partition was removed at the beginning of the session, and a mouse was then placed in the light compartment. The mouse could then move freely between the two compartments, and its behavior was recorded over 700 s. Since the latency to enter and the number of entries into the dark compartment should each reflect avoidance, both were measured from the recorded video. The latency ceiling was fixed at 700 s.

#### Fear-conditioning test

To test sex difference in associative learning, and how it may contribute to any observed differences in avoidance, we performed a fear-conditioning test based on a protocol [28].

Twelve male and 31 female (8 proestrus, 9 estrus, 10 diestrus, 4 nd) mice were used. All stimulus presentation was computer-controlled (Image FZC, O’Hara & Co., Tokyo, Japan). This test comprised of four sessions: training, contextual fear-conditioning (CXT), pre-auditory-cued fear-conditioning (pre-AUD), and auditory-cued fear-conditioning (AUD). The training session was carried out on day 1, and the remaining three sessions were conducted the next day.

In the training session, mice learned the association between a foot-shock and an accompanying cue. Mice were placed individually in an operant chamber (width = 120 mm, height and depth = 110 mm; O’Hara & Co.). After placement, contextual (a mixture of implicit cues, such as odor, field-view, and sound, in the chamber) cues were presented for 30 s. The last 2 s of auditory (10 kHz, 75 dB) cue presentation were accompanied by a 0.75 mA foot-shock delivered from stainless steel bars on the floor. After the foot-shock, the mice were returned to their home cages. After the training session, it was expected that mice could anticipate the upcoming foot-shock whenever either cue was presented.

Twenty-four hours after the training session, mice were subjected to the CXT session. They were re-exposed to the same chamber and the same contextual cues, as in the training session. They spent 180 s in this chamber with neither an auditory cue nor a foot-shock.

Two hours later, the same mice were subjected to the pre-AUD and AUD sessions. They were placed in a novel chamber, which formed a triangular prism (side = 110 mm, height = 120 mm, O’Hara & Co.), for 360 s. To mask the olfactory cues, the chamber was cleaned with sodium hypochlorite before and after each use. The mice were re-exposed, 180 s after placement, to the same auditory cue as in the training session (AUD). To ensure that the mice were subjected to the AUD session without generalized contextual fear carried over from the preceding session, we also measured their response in the first 180 s (pre-AUD).

We measured freezing as a conditioned response in each mouse, as it is recognized as a behavioral index of associative learning. Behavior in the chamber was recorded at one frame per second, and a freezing response was defined as any instance when a difference of pixel intensities between two successive frames was less than 30% (Image FZC).

#### Light/dark and light/light test

In the active and passive avoidance tests, the perception of potential threats prompts the subsequent avoidance. We expected that if the perception of potential threats was influenced by a sex difference in both types of avoidance, the difference would be diminished in the absence of the perceived threat. Therefore, we carried out the light/dark (L/D) and light/light (L/L) tests as additional contexts. In the L/D and L/L tests, mice did not encounter the threat or the threat-predicting cue, respectively. The sessions and the measured behavior were the same as in the passive avoidance test (see above).

In the L/D test, 12 mice (6 male and 6 female) were used. The chamber was the same as in the passive avoidance test, except that the foot-shock was never applied. In the L/L test, 36 mice (6 male and 30 female) were used. The chamber was the same as in the active avoidance test, except that the auditory stimulus was never presented.

#### Tail suspension test

We tested whether a sex difference arises in the escape component using the tail suspension test (TST) and the forced swim test (FST). In both tests, mice faced an imminent threat, rather than a potential one.

The TST was used to examine 12 male and 17 female (5 proestrus, 6 estrus, 4 diestrus, 2 nd) mice. The experimental procedure was based on previous studies 29–31]. Briefly, mice were suspended by their tails from a fixture 35 cm above the floor for 360 s. Their behavior was recorded and analyzed by motion analysis software (Image FZC).

Since immobility indicates how individuals attempt to escape behavior from an imminent threat [13, 14, 32–34], we measured the immobility of the trunk, excluding slight movements of the limbs and tail, and calculated the total time spent immobile (immobility time) for data analysis. This procedure was conducted daily on 2 consecutive days.

#### Forced swim test

The FST was used to examine 12 male and 18 female (4 proestrus, 4 estrus, 7 diestrus, and 3 nd) mice. The experimental procedure was based on previous studies [29–31]. For 360 s, mice were placed in a glass beaker (diameter = 135 mm, height = 200 mm; Hario Glass Co., Tokyo, Japan), which was filled to a height of 130 mm with water at 25 ± 1 °C. Behavior in the pool was recorded and analyzed using Image FZC software. Immobility was measured as passive floating with slight movements of the limbs and tail, and the immobility time was calculated. This procedure was conducted daily on 2 consecutive days.

#### Measurement of basal activity in familiar home cage

We checked whether a sex difference arises in the basal activity in the familiar home cage in the absence of any threat. This test was used to examine 8 male and 8 female mice. Each mouse was housed in a separate standard laboratory home cage (width = 203 mm, height = 118 mm, depth = 133 mm) for 3 days. On the 4th day, their activity was measured for 1 min with a camera (1.3 million pixels, viewing angle of 78°, 30 frames/s). The mean velocity, total travel distance, and distance from the center were calculated from the track point on the body by Motion Analyzer (version 1.4.21.0, Keyence Co. Ltd., Osaka, Japan).

### Data analysis

In all tests, we recorded 1–3 behavioral measures per test for each mouse, and then calculated the group mean with a 95% confidence interval (CI) for each of the following groups: males, females, and females in each estrous phase. We excluded subjects that exceeded 2 standard deviations of the group mean. For statistical analysis, females in specific estrous phases and all females group were treated as independent groups whenever either was compared with males. We performed Shaffer’s modified sequentially rejective Bonferroni procedure as a multiple comparison whenever we found statistical significance in 1- or 2-factorial analysis of variance (ANOVA). Alpha was set at 0.05. We calculated the effect size [Pearson’s *R* on *t* test and the multiple comparisons, generalized eta-squared (η_*G*_^2^) on ANOVA] where η_*G*_^2^ is a recommended effect size statistic for various experimental designs [35], including the mixed design used in the present study. These effect size statistics are available indices that are standardized to quantify the practical magnitude of the relationship between independent and dependent variables, independent of the sample size, and thereby, enable results to be compared with other studies [35]. The magnitude of the effect size was interpreted as either small, medium, or large in accordance with the standard guidelines [36, 37]. The sex ratio (SR) = male/female * 100 was calculated from the group mean of males and that of females in each test. Statistical analysis was done by customized codes in MATLAB (MathWorks Inc., MA, US).

In the active avoidance test, we calculated the avoidance rate in 5 bins of 20 trials for each mouse, and the group mean for males, females, and females in each estrous phase. We performed a 2-way [sex (males, females) × bin (1st, 2nd, 3rd, 4th, 5th)] ANOVA for the avoidance rate. To compare males with females in each estrous phase, another 2-way [sex (males, proestrus, estrus, diestrus) × bin (1st, 2nd, 3rd, 4th, 5th)] ANOVA was performed.

In the passive avoidance test, we calculated the latency to enter and the number of entries into the dark compartment for each mouse in the test session, and then, the group mean for males, females, and females in each estrous phase. We performed a 2-sample *t* test (males vs. females) and a 1-way [sex (males, proestrus, estrus, diestrus)] ANOVA for latency. A 2-sample *t* test (males vs. females) for the number of transitions was also performed.

In the fear-conditioning test, we calculated the percentage of time spent freezing (freezing rate) for each mouse in each session, and then, the group mean for males, females, and females in each estrous phase. We performed a 2-way [sex (males or females) × session (training, CXT, pre-AUD, AUD)] ANOVA for the freezing rate. To compare males with females in each estrous phase, another 2-way [sex (males, proestrus, estrus, diestrus) × session (training, CXT, pre-AUD, AUD)] ANOVA was performed.

As in the passive avoidance test, we performed a 2-sample *t* test (males vs. females) for latency in the L/D and L/L tests.

In both the TST and FST, we calculated the immobility time for each mouse on both days. The group mean was then calculated for males, females, and females in each estrous phase. We performed 2-way [sex (males or females) × day (1st or 2nd)] ANOVA for immobility time. To compare males with females in each estrous phase, another 2-way [sex (males, proestrus, estrus, diestrus) × day (1st or 2nd)] ANOVA was performed.

For measurement of the basal activity in the familiar home cage, mean velocity, total travel distance, and distance from the center of the cage were measured. After the individual mean and the group mean were calculated, a 2-sample *t* test (males vs. females) was performed for each motion parameter.

## Results

### Two-way active avoidance test

**Fig. 1:**
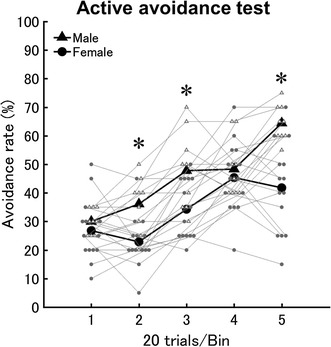
Sex differences in mobility in the active avoidance test. *X* and *Y axis* indicates bins per 20 trials and avoidance rate, respectively. *White triangles* and *gray circle* indicates either males (n = 9) or females (n = 14). The mean avoidance rate of males and females were indicated by *black triangles* and *circles*, respectively. *Asterisks* indicate bins showing significant sex difference

The 2-way ANOVA comparing males to females for the active avoidance rate showed a significant interaction between sex and bin (each 20 trials) (*F*_4,104_ = 4.20, *p* < 0.05, η_*G*_^2^ = 0.07). The avoidance rate between the sexes within each bin was compared. Although the avoidance rate in the initial bin was similar between the sexes, in most of the subsequent bins, males exhibited a significantly higher avoidance rate than females (*p* < 0.05) (Fig. 1).

When we compared the avoidance rate between the bins within each sex, males exhibited a steeper learning curve than females (Fig. 1). In males, the avoidance rate for bin 5 was significantly higher than all of the previous bins (*p* < 0.05). Similarly, bins 3 and 4 showed significantly higher avoidance rates than observed during bins 1 and 2 (*p* < 0.05). By contrast, in females, the avoidance rate during bin 5 was only significantly higher than bins 1 and 2 (*p* < 0.05). Although bin 4 showed a significantly higher avoidance rate than the previous 3 bins (*p* < 0.05), the avoidance rate during bin 3 was only higher than bin 2 (*p* < 0.05).

The 2-way ANOVA comparing males to females in different estrous cycle for the active avoidance rate showed a significant interaction between sex/phase of estrous cycle and bin (*F*_12,96_ = 2.28, *p* < 0.05, η_*G*_^2^ = 0.12). Significant simple main effects of sex/phase of estrous cycle were observed at bins 2 and 5 (Additional file 1: Figure S1). Specifically, females in estrus exhibited a significantly lower avoidance rate than males in both bins (*p* < 0.05). Similarly, females in diestrus and proestrus showed a significantly lower avoidance rate than males in bins 2 and 5, respectively (*p* < 0.05).

The learning curve of the avoidance also significantly differed between males and females in each estrous phase (Additional file 1: Figure S1). Although males showed a steep learning curve as described above, females in proestrus exhibited an invariant avoidance rate across the bins. For females in estrus, only bin 4 showed a significantly higher avoidance rate than bins 2 and 3. Similarly, for females in diestrus the avoidance rate during bins 4 and 5 was significantly higher than the rate during bins 1 and 2.

### Passive avoidance test

**Fig. 2:**
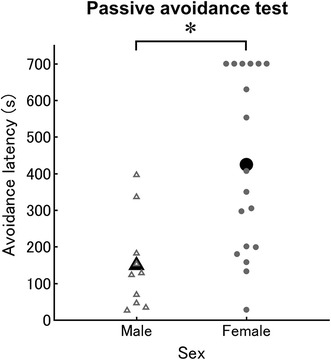
Sex differences in immobility in the passive avoidance test. *X* and *Y axis* indicates sex and avoidance latency, respectively. *White triangles* and *gray circles* indicate scores in individuals in males (n = 10) and females (n = 18), respectively. The mean avoidance rate in males and females were indicated by a *black triangle* and a *circle*, respectively. *p < 0.05

Males exhibited a significantly higher number of entries into the dark compartment than females (2-sample *t* test; *t*_25_ = 3.13, *p* < 0.05, Pearson’s *R* = 0.53, SR = 307.69%). When we compared the latency to enter the dark chamber between the sexes, we found that males also had significantly shorter latencies than females (2-sample *t* test; *t*_25_ = 3.74, *p* < 0.05, Pearson’s *R* = 0.60, SR = 33.34%) (Fig. 2).

We also found a significant difference in latency between males and females in different phases of the estrous cycle (1-way ANOVA; *F*_3,22_ = 6.08, *p* < 0.05, η_*G*_^2^ = 0.45) (Additional file 2: Figure S2). The latency for females in each estrous group decreased in the following order: proestrus, estrus, and diestrus. Females in proestrus and estrus showed significantly longer latencies than males (*p* < 0.05, Pearson’s *R* = 0.60 and 0.48, respectively) (Additional file 2: Figure S2).

### Fear-conditioning test

**Fig. 3:**
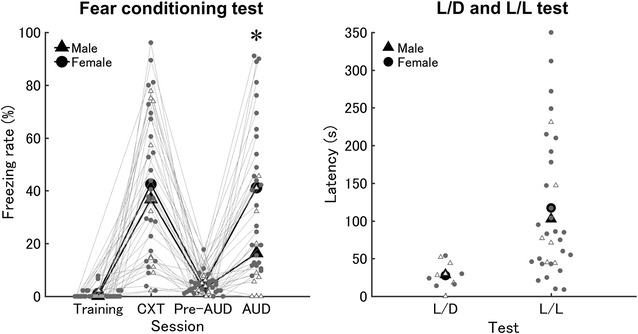
Sex difference in risk perception in cued fear-conditioning test, and the absence of sex differences in mobility in the L/D and L/L tests. Mean freezing rate in males and females in the Training, CXT, pre-AUD, and AUD sessions in the fear-conditioning test (*left panel*). *X* and *Y axis* indicates session name and freezing rate, respectively. *White triangles* and *gray circles* indicate scores in individuals in males (n = 11) and females (n = 27), respectively. Mean freezing rate in males and females were indicated by *black triangles* and *circles*, respectively. An *asterisk* indicates the session showing significant sex difference. Mean avoidance latencies in males, females in the L/D and L/L tests (*right panel*). *X* and *y axis* indicates test name and avoidance latency, respectively. *White triangles* and *gray circles* indicate scores in individuals in males (n = 6 for L/D; n = 6 for L/L) and females (n = 5 for L/D; n = 27 for L/L), respectively. The mean latency in males and females were indicated by a *black triangle* and a *circle*, respectively

The 2-way ANOVA for the freezing rate showed a significant interaction between sex and session, although the size of the effect was small (*F*_3,108_ = 3.34, *p* < 0.05, η_*G*_^2^ = 0.05) (Fig. 3, left panel).

When we compared the freezing rate between sexes within each session, we found a significant sex difference only in the AUD session (*p* < 0.05, Pearson’s *R* = 0.40) (Fig. 3, left panel). The SRs during training, CXT, pre-AUD, and AUD were 63.47, 86.95, 21.05, and 39.73%, respectively. We also compared the freezing rate within females between sessions. The freezing rate in the AUD session was significantly higher than in the training and pre-AUD sessions (*p* < 0.05, Pearson’s *R* = 0.75 and 0.57, respectively). Similar results were observed in the CXT session (*p* < 0.05, Pearson’s *R* = 0.73 and 0.70, respectively), with no significant difference between the AUD and CXT sessions (*p* > 0.05, Pearson’s *R* = 0.01). In contrast, the male freezing rate in the CXT session was significantly higher than in any other session (*p* < 0.05, Pearson’s *R* = 0.70, 0.67, and 0.42 for training, pre-AUD and AUD, respectively), whereas the AUD session showed no significant difference when compared with the training and pre-AUD sessions (*p* > 0.05, Pearson’s *R* = 0.62 and 0.55, respectively). When we compared the freezing rate between males and females in each estrous phase, neither a significant main effect of sex/phase of estrous cycle (*F*_3,30_ = 1.30, *p* > 0.05, η_*G*_^2^ = 0.04) nor a significant interaction between group and session (*F*_9,90_ = 1.16, *p* > 0.05, η_*G*_^2^ = 0.09) was observed (Additional file 3: Figure S3).

We confirmed that mice could participate in the AUD session without a generalization of contextual fear memory from the preceding CXT session, because the freezing rate in neither males nor females differed between the training and pre-AUD sessions (see above). In addition, each contextual and auditory cue should independently induce the freezing response, because a contextual cue was sufficient for the mice to perceive the foot-shock in the CXT session. Likewise, an auditory cue was sufficient for foot-shock perception in the AUD session.

Although we found a significant interaction between sex and session, the magnitude of the effect of sex in this test was small according to the guideline (see “Methods”), even when it was compared to those in both avoidance tests. Thus, we concluded that the sex difference in associative learning was small.

### L/D and L/L test

No significant sex difference was observed in either the L/D (2-sample *t* test; *t*_31_ = 0.35, *p* > 0.05, Pearson’s *R* = 0.05, SR = 103.88%) or the L/L tests (2-sample *t* test; *t*_9_ = 0.10, *p* > 0.05, Pearson’s *R* = 0.02, SR = 87.41%) (Fig. 3, right panel).

### Tail suspension test

**Fig. 4:**
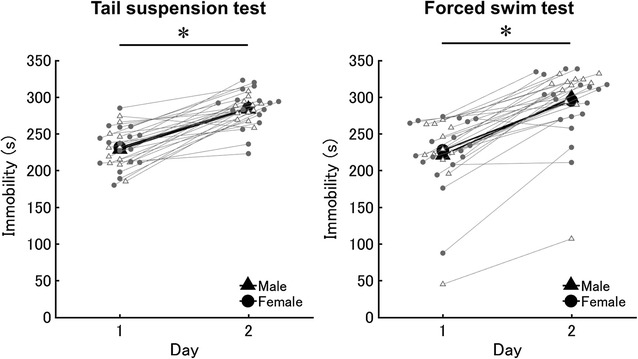
Absence of sex differences in responses in the TST and FST. Mean immobility time in males and females across day 1 and 2 in the TST (*left panel*). Mean immobility time in males and females across day 1 and 2 in the FST (*right panel*). *X* and *Y axis* indicates day and immobility time, respectively. *White triangles* and *gray circles* indicate scores in individuals in males (n = 12 for TST; n = 12 for FST) and females (n = 17 for TST; n = 18 for FST), respectively. The mean immobility in males and females were indicated by *black triangles* and *circles*, respectively. Each *asterisk* indicates significant increasing the immobility from day 1 to 2

For immobility during the TST, neither a significant interaction between sex and day (*F*_1,26_ = 0.01, *p* = 0.92, η_*G*_^2^ = 0.00) nor a significant main effect of sex (*F*_1,26_ = 0.37, *p* = 0.55, η_*G*_^2^ = 0.01) was observed (Fig. 4, left panel). The SR values were 98.35 and 98.25% on days 1 and 2, respectively. However, the immobility was significantly higher for both sexes on day 2 than on day 1 (main effect of day: *F*_1,27_ = 96.15, *p* < 0.05, η_*G*_^2^ = 0.58) (Fig. 4, left panel). This effect was also observed when we compared males with females in each estrous phase (main effect of day: *F*_1,23_ = 65.83, *p* < 0.01, η_*G*_^2^ = 0.49). Neither a significant main effect of sex/phase of estrous cycle (*F*_3,23_ = 0.08, *p* = 0.97, η_*G*_^2^ = 0.01) nor a significant interaction between sex/phase of estrous cycle and day (*F*_3,23_ = 0.52, *p* = 0.67, η_*G*_^2^ = 0.02) was observed (Additional file 4: Figure S4A, B).

### Forced swim test

Results analyzing the immobility in the FST were similar to those in the TST. There was neither a significant interaction between sex and day (*F*_1,25_ = 1.01, *p* = 0.32, η_*G*_^2^ = 0.01) nor a significant main effect of sex (*F*_1,25_ = 0.51, *p* = 0.48, η_*G*_^2^ = 0.01) (Fig. 4, right panel). The SR values were 99.78 and 103.80% on days 1 and 2, respectively, and immobility increased significantly on day 2 in both sexes (main effect of day: *F*_1,25_ = 149.20, *p* < 0.05, η_*G*_^2^ = 0.69) (Fig. 4, right panel). This effect was also observed when we compared males with females in each estrous phase (main effect of day: *F*_1,21_ = 122.86, *p* < 0.05, η_*G*_^2^ = 0.51) (Fig. 4, right panel). Although there was no significant main effect of sex/phase of estrous cycle (*F*_3,21_ = 0.61, *p* = 0.62, η_*G*_^2^ = 0.06), there was an apparent trend toward significance for the interaction between sex/phase of estrous cycle and day (*F*_3,21_ = 2.56, *p* = 0.08, η_*G*_^2^ = 0.07) (Additional file 4: Figure S4C, D).

### Measurement of basal activity in familiar home cage

Each motion parameter (mean ± CI) in a familiar home cage was comparable between sexes. As such, no significant difference was observed in the mean velocity (male = 43.76 ± 7.91 mm/s vs. female = 38.60 ± 7.48 mm/s, *p* > 0.05, Pearson’s *R* = 0.23, SR = 113.54%), total distance travelled (male = 2371.42 ± 457.74 mm vs. female = 1795.86 ± 615.33 mm, *p* > 0.05, Pearson’s *R* = 0.36, SR = 132.05%), or distance from the center of the cage (male = 69.57 ± 7.37 mm vs. female = 75.54 ± 4.71 mm, *p* > 0.05, Pearson’s *R* = 0.32, SR = 92.07%).

## Discussion

In this study, we investigated sex-mediated responses in behaviors under a variety of risks. Since a behavior is composed of a set of simpler responses toward a specific scene, scene segmentation, which involves breaking down a general context to each scene, would be an efficient way to extract subtle phenotypes in a behavior and their occurrence conditions. In line with this, we ran a battery of behavior tests modeling discrete types of risks. We hypothesized that avoidance response to an escapable risk would diverge between sexes. In the present battery of tests, avoidance was further segmented into active and passive sub-components, which were tested in the two-way active avoidance test and the passive avoidance test, respectively. Then, the fear-conditioning test was used to determine whether sex differences arise in the formation of associative learning alone, since it is necessary for responding to a predicted risk, and thereby required for learned avoidance behaviors [13]. The L/D and L/L tests were run as additional scenes in which the escapable risk and the risk-predicting cue were uncoupled. To check if sex differences arise in active and passive responding to an aversive, inescapable scene, the forced-swim test and tail-suspension test were done. Basal activity in a home cage was also compared between sexes.

Sex differences were shown to occur in the active as well as the passive avoidance tests. In general, the evidence suggested that males responded to a risk with higher mobility while females were less mobile after predicting an impending foot-shock from an associated cue. More specifically, in the active avoidance test, males exhibited more transitions from the shock compartment to the safe compartment, as the avoidance rate in the last bin (trials 81–100) for males was nearly 150% of that shown by females. Similarly, in the passive avoidance test, transition to the dark compartment in males was almost 300% of that in females. Likewise, the latency of entry into the dark compartment for males was 30% of that of females. These differences could not be attributed to a difference in basic locomotor activity, because all motion parameters measured in the familiar home cage were comparable between sexes. Thus, we conclude that males express a higher exploratory avoidance than females in response to an escapable risk. These differences in mobility present an opportunity to learn to avoid the threat under the active avoidance test, but increase males’ exposure to threat in the passive avoidance test.

When we consider the sexually different cognitive processing underlying the avoidance responses, we can assume that it requires a process from the perception of a potential risk to the subsequent execution of avoidance. Indeed, the perception of a potential risk relied on associative learning between a risk and a prediction cue in both types of avoidance tests. The sex difference in the active avoidance test was primarily observed in the later bins where the association between the cue and the threat was fully formed. In the fear-conditioning test, associative learning occurred despite an inability to carry out subsequent avoidance behavior. In this test, the higher freezing rates in females shown in auditory-cued fear conditioning might partially contribute to their immobility during avoidance. This is also supported by the results of the L/D and L/L tests, and is consistent with previous studies, which found no sex difference in tests with a lack of associative learning to perceive a potential risk, such as the elevated plus maze test [38, 39]. In the scenes with inescapable risks, sex differences disappeared as shown in the TST and the FST.

With regard to the neural mechanisms that induce these differences between sexes, the projection from the lateral habenula to the rostromedial tegmental nucleus in the midbrain is a possible candidate. The lateral habenula encodes both active and passive avoidance behavior associated with negative reward [40], and a sex difference has been observed in this region [41–43]. In addition, the lateral habenula shows a high expression of the estrogen receptor [43]. Estrogen facilitates anxiety-like behavior in a variety of tests including not only auditory-cued fear conditioning and passive avoidance tests, but also the elevated plus maze and open field test [44–46]. In addition to these anxiogenic effects, estrogen also decreases locomotor activity, such as the number of rotations in a running wheel or the travel distance in a new environment [45, 46]. Thus, estrogen in the lateral habenula may account for the lower mobility in both types of avoidance in females in the estrus and proestrus phases.

In summary, the present study indicated that subtle divergences between sexes in sequentially varying behavior under risky contexts were successfully extracted by scene segmentation. We now know that sex-dependent divergences occur in the degree of locomotion in avoidance behaviors. Nevertheless, significant components would be still latent in a considerable number of behaviors. Hence, further study would be required for extracting them in order to comprehensively understand a behavior. For a general point of view to our behavior, we concluded the computational analysis of behavior needed to be established to understand our social behaviors like sex differences.

## Additional files

**Additional file 1: Figure S1.** Sexually different mobility in the active avoidance test between male and females in each estrous phase. X and Y axis indicates bins per 20 trials and avoidance rate, respectively. (A) White triangles and dark gray circles, medium gray squares, and light gray diamonds indicates males (n = 9) or females in proestrus (n = 3), estrus (n = 5), and diestrus (n = 6). (B) The mean avoidance rate of males and females in each estrous phase were indicated by white triangles and dark gray circles, medium squares, and light diamonds, respectively. An asterisk and a dagger indicates the bin showing significant sex difference between male and females except in the proestrus phase, and that between male and females except in the diestrus phase, respectively.

**Additional file 2: Figure S2.** Sexually different immobility in the active avoidance test between male and females in each estrous phase. X and Y axis indicates sex and avoidance latency, respectively. White triangles, dark gray circles, medium gray squares, and light gray diamonds indicate scores in individuals in males (n = 10) and females in proestrus (n = 3), estrus (n = 8), and diestrus (n = 6) phase respectively. The mean avoidance rate in males and females in each estrus phase were indicated by a black triangle, circle, square, and diamond, respectively. Two asterisks indicate bins showing significant sex difference between male and females in proestrus, and male and females in estrus phase, respectively.

**Additional file 3: Figure S3.** Sexually different risk perception in cued fear-conditioning test between males and females in each estrous phase. X and Y axis indicates session name and freezing rate, respectively. (A) White triangles, dark gray circles, medium gray squares, and light gray diamonds indicate scores in individuals in males (n = 11) and females in proestrus (n = 8), estrus (n = 8), and diestrus (n = 7), respectively. (B) Mean freezing rate in males and females in each estrus phase were indicated by white triangles, dark gray circles, medium gray squares, and light gray diamonds respectively.

**Additional file 4: Figure S4.** Absence of sex differences between males and females in each estrous phase in responses in the TST (A, B) and FST (C, D). X and Y axis indicates day and immobility time, respectively. (A) White triangles, dark gray circles, medium gray squares, and light gray diamonds indicate scores in individuals in males (n = 12) and females in proestrus (n = 5), estrus (n = 6), and diestrus (n = 4) phase, respectively. (B) The mean immobility in males and females in each estrus phase were indicated by black triangles, dark gray circles, medium gray squares, and light gray diamonds, respectively. (C) Scores in individuals in males (n = 12) and females in proestrus (n = 4), estrus (n = 4) and diestrus (n = 7) were indicated by the same maker types with (A). (D) The mean immobility in males and females in each estrus phase were indicated by the same marker types with (B), respectively. Each asterisk indicates significant increasing the immobility from day 1 to 2.

## References

[1] Cahill L (2006). Why sex matters for neuroscience. Nat Rev Neurosci.

[2] Kimchi T, Xu J, Dulac C (2007). A functional circuit underlying male sexual behaviour in the female mouse brain. Nature.

[3] Pasterski V, Hindmarsh P, Geffner M, Brook C, Brain C, Hines M (2007). Increased aggression and activity level in 3- to 11-year-old girls with congenital adrenal hyperplasia (CAH). Horm Behav.

[4] Ronkainen H, Ylönen H (1994). Behaviour of cyclic bank voles under risk of mustelid predation: do females avoid copulations?. Oecologia.

[5] Tan J, Ma Z, Gao X, Wu Y, Fang F (2011). Gender difference of unconscious attentional bias in high trait anxiety individuals. PLoS ONE.

[6] Forger NG, de Vries GJ (2010). Cell death and sexual differentiation of behavior: worms, flies, and mammals. Curr Opin Neurobiol.

[7] Benjamini Y, Lipkind D, Horev G, Fonio E, Kafkafi N, Golani I (2010). Ten ways to improve the quality of descriptions of whole-animal movement. Neurosci Biobehav Rev.

[8] Benjamini Y, Fonio E, Galili T, Havkin GZ, Golani I (2011). Quantifying the buildup in extent and complexity of free exploration in mice. Proc Natl Acad Sci USA.

[9] Burgos-Artizzu XP, Dollár P, Lin D, Anderson DJ, Perona P. Social behavior recognition in continuous video. In: 2012 IEEE conference on computer vision and pattern recognition (CVPR). Piscataway: IEEE; 2012. p. 1322–9.

[10] Neuberg SL, Kenrick DT, Schaller M (2011). Human threat management systems: self-protection and disease avoidance. Neurosci Biobehav Rev.

[11] Topál J, Csányi V (1994). The effect of eye-like schema on shuttling activity of wild house mice (*Mus musculus domesticus*): context-dependent threatening aspects of the eyespot patterns. Anim Learn Behav.

[12] Colwill RM, Creton R (2011). Imaging escape and avoidance behavior in zebrafish larvae. Rev Neurosci.

[13] Cryan JF, Holmes A (2005). The ascent of mouse: advances in modelling human depression and anxiety. Nat Rev Drug Discov.

[14] Jesuthasan S (2012). Fear, anxiety, and control in the zebrafish. Dev Neurobiol.

[15] Richter J, Hamm AO, Pane-Farre CA, Gerlach AL, Gloster AT, Wittchen HU (2012). Dynamics of defensive reactivity in patients with panic disorder and agoraphobia: implications for the etiology of panic disorder. Biol Psychiatry.

[16] Rodgers RJ, Cao BJ, Dalvi A, Holmes A (1997). Animal models of anxiety: an ethological perspective. Braz J Med Biol Res.

[17] Rosen JB, Schulkin J (1998). From normal fear to pathological anxiety. Psychol Rev.

[18] Schlund MW, Cataldo MF (2010). Amygdala involvement in human avoidance, escape and approach behavior. Neuroimage.

[19] Korte SM, De Boer SF, Bohus B (1999). Fear-potentiation in the elevated plus-maze test depends on stressor controllability and fear conditioning. Stress.

[20] Barkus C, McHugh SB, Sprengel R, Seeburg PH, Rawlins JNP, Bannerman DM (2010). Hippocampal NMDA receptors and anxiety: at the interface between cognition and emotion. Eur J Pharmacol.

[21] Dohanich G (2003). Ovarian steroids and cognitive function. Curr Dir Psychol Sci.

[22] Frohlich J, Morgan M, Ogawa S, Burton L, Pfaff D (2002). Statistical analysis of hormonal influences on arousal measures in ovariectomized female mice. Horm Behav.

[23] Kuriyama H, Shibasaki T (2004). Sexual differentiation of the effects of emotional stress on food intake in rats. Neuroscience.

[24] Miller G, Tybur JM, Jordan BD (2007). Ovulatory cycle effects on tip earnings by lap dancers: economic evidence for human estrus?. Evol Hum Behav.

[25] Caligioni CS (2009). Assessing reproductive status/stages in mice. Curr Protoc Neurosci.

[26] Chang T, Meyer U, Feldon J, Yee BK (2007). Disruption of the US pre-exposure effect and latent inhibition in two-way active avoidance by systemic amphetamine in C57BL/6 mice. Psychopharmacology.

[27] Iso H, Simoda S, Matsuyama T (2007). Environmental change during postnatal development alters behaviour, cognitions and neurogenesis of mice. Behav Brain Res.

[28] Nakazawa T, Komai S, Watabe AM, Kiyama Y, Fukaya M, Arima-Yoshida F (2006). MR2B tyrosine phosphorylation modulates fear learning as well as amygdaloid synaptic plasticity. EMBO J.

[29] Kromer SA, Kessler MS, Milfay D, Birg IN, Bunck M, Czibere L (2005). Identification of glyoxalase-I as a protein marker in a mouse model of extremes in trait anxiety. J Neurosci.

[30] Jacobsen JP, Weikop P, Hansen HH, Mikkelsen JD, Redrobe JP, Holst D (2008). SK3K+ channel-deficient mice have enhanced dopamine and serotonin release and altered emotional behaviors. Genes Brain Beha.

[31] McEuen JG, Semsar KA, Lim MA, Bale TL (2009). Influence of sex and corticotropin-releasing factor pathways as determinants in serotonin sensitivity. Endocrinology.

[32] Imai S, Mamiya T, Tsukada A, Sakai Y, Mouri A, Nabeshima T (2012). Ubiquitin-specific peptidase 46 (Usp46) regulates mouse immobile behavior in the tail suspension test through the GABAergic system. PLoS ONE.

[33] Adamec R, Head D, Blundell J, Burton P, Berton O (2006). Lasting anxiogenic effects of feline predator stress in mice: sex differences in vulnerability to stress and predicting severity of anxiogenic response from the stress experience. Physiol Behav.

[34] Adamec R, Head D, Soreq H, Blundell J (2008). The role of the read through variant of acetylcholinesterase in anxiogenic effects of predator stress in mice. Behav Brain Res.

[35] Olejnik S, Algina J (2003). Generalized eta and omega squared statistics: measures of effect size for some common research designs. Psychol Methods.

[36] Cohen J (1988). Statistical power analysis for the behavioral sciences.

[37] Field A (2009). Discovering statistics using SPSS.

[38] Chikahisa S, Sano A, Kitaoka K, Miyamoto K, Sei H (2007). Anxiolytic effect of music depends on ovarian steroid in female mice. Behav Brain Res.

[39] O’Leary TP, Gunn RK, Brown RE (2013). What are we measuring when we test strain differences in anxiety in mice?. Behav Genet.

[40] Stamatakis AM, Stuber GD (2012). Activation of lateral habenula inputs to the ventral midbrain promotes behavioral avoidance. Nat Neurosci.

[41] Brown LL, Siegel H, Etgen AM (1996). Global sex differences in stress-induced activation of cerebral metabolism revealed by 2-deoxyglucose autoradiography. Horm Behav.

[42] Lonstein JS, De Vries GJ (1999). Sex differences in the parental behaviour of adult virgin prairie voles: independence from gonadal hormones and vasopressin. J Neuroendocrinol.

[43] Yokosuka M, Okamura H, Hayashi S (1997). Postnatal development and sex difference in neurons containing estrogen receptor-alpha immunoreactivity in the preoptic brain, the diencephalon, and the amygdala in the rat. J Comp Neurol.

[44] Marcondes FK, Miguel KJ, Melo LL, Spadari-Bratfisch RC (2001). Estrous cycle influences the response of female rats in the elevated plus-maze test. Physiol Behav.

[45] Morgan MA, Pfaff DW (2001). Effects of estrogen on activity and fear-related behaviors in mice. Horm Behav.

[46] Morgan MA, Pfaff DW (2002). Estrogen’s effects on activity, anxiety, and fear in two mouse strains. Behav Brain Res.

