# Supplementary figures and images for: Sex differences in avoidance behavior after perceiving potential risk in mice
Source: Behav Brain Funct. 2017 May 5;13:9. doi: 10.1186/s12993-017-0126-3 (PMC5420094; doi:10.1186/s12993-017-0126-3)

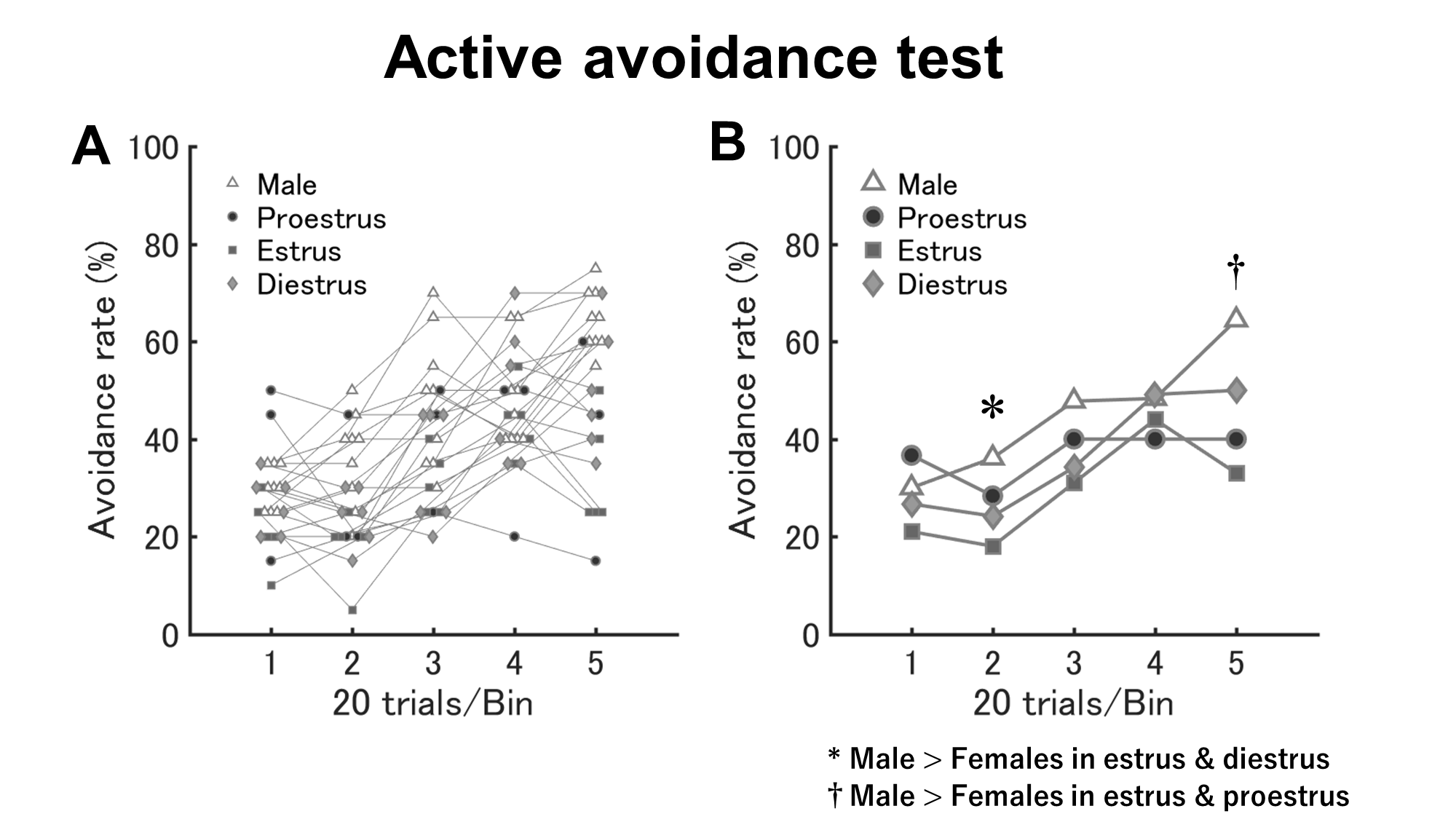

Supplement: Supplementary file 1 — Additional file 1: Figure S1. Sexually different mobility in the active avoidance test between male and females in each estrous phase. X and Y axis indicates bins per 20 trials and avoidance rate, respectively. (A) White triangles and dark gray circles, medium gray squares, and light gray diamonds indicates males (n = 9) or females in proestrus (n = 3), estrus (n = 5), and diestrus (n = 6). (B) The mean avoidance rate of males and females in each estrous phase were indicated by white triangles and dark gray circles, medium squares, and light diamonds, respectively. An asterisk and a dagger indicates the bin showing significant sex difference between male and females except in the proestrus phase, and that between male and females except in the diestrus phase, respectively. [file 12993_2017_126_MOESM1_ESM.tif]

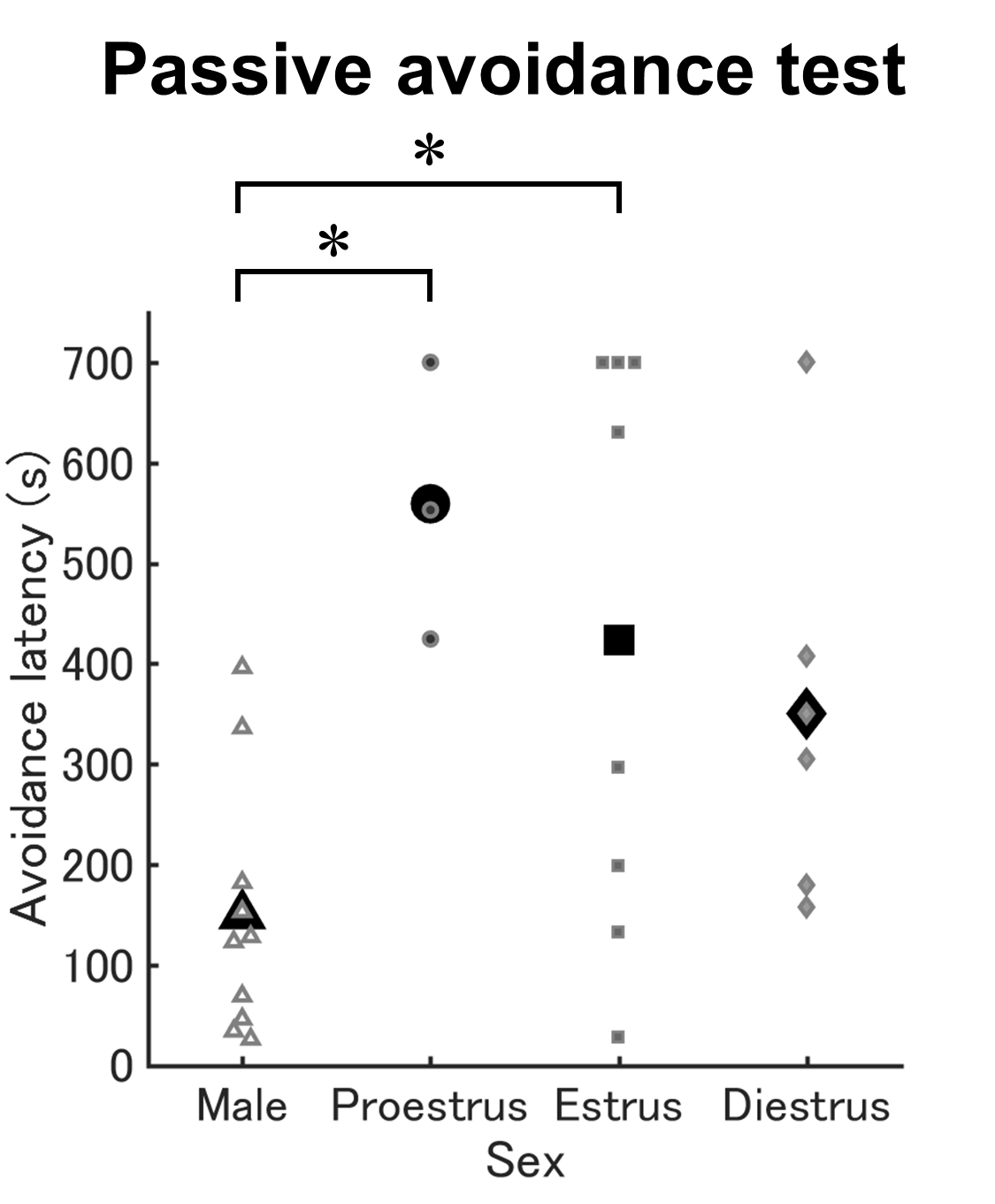

Supplement: Supplementary file 2 — Additional file 2: Figure S2. Sexually different immobility in the active avoidance test between male and females in each estrous phase. X and Y axis indicates sex and avoidance latency, respectively. White triangles, dark gray circles, medium gray squares, and light gray diamonds indicate scores in individuals in males (n = 10) and females in proestrus (n = 3), estrus (n = 8), and diestrus (n = 6) phase respectively. The mean avoidance rate in males and females in each estrus phase were indicated by a black triangle, circle, square, and diamond, respectively. Two asterisks indicate bins showing significant sex difference between male and females in proestrus, and male and females in estrus phase, respectively. [file 12993_2017_126_MOESM2_ESM.tif]

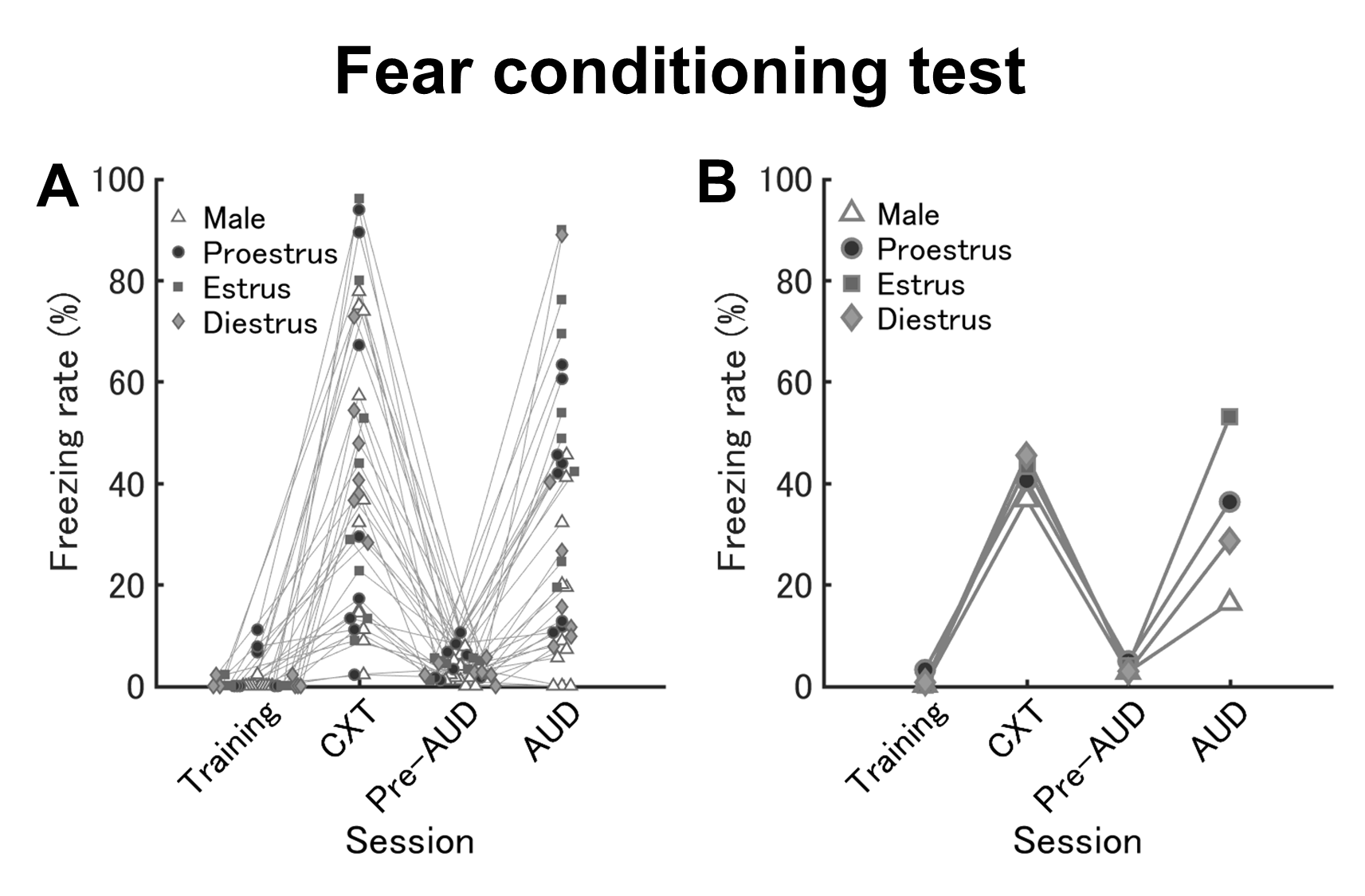

Supplement: Supplementary file 3 — Additional file 3: Figure S3. Sexually different risk perception in cued fear-conditioning test between males and females in each estrous phase. X and Y axis indicates session name and freezing rate, respectively. (A) White triangles, dark gray circles, medium gray squares, and light gray diamonds indicate scores in individuals in males (n = 11) and females in proestrus (n = 8), estrus (n = 8), and diestrus (n = 7), respectively. (B) Mean freezing rate in males and females in each estrus phase were indicated by white triangles, dark gray circles, medium gray squares, and light gray diamonds respectively. [file 12993_2017_126_MOESM3_ESM.tif]

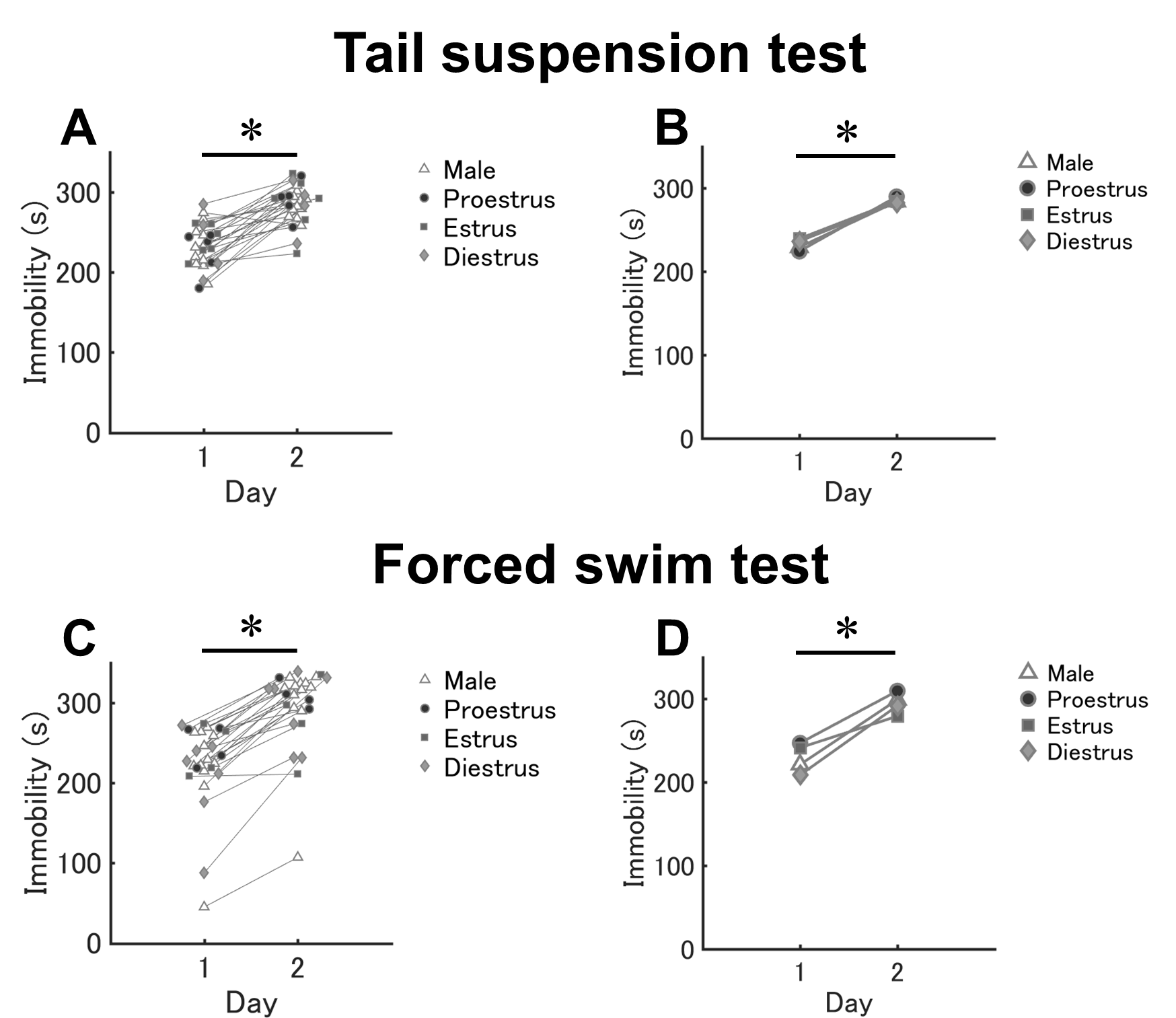

Supplement: Supplementary file 4 — Additional file 4: Figure S4. Absence of sex differences between males and females in each estrous phase in responses in the TST (A, B) and FST (C, D). X and Y axis indicates day and immobility time, respectively. (A) White triangles, dark gray circles, medium gray squares, and light gray diamonds indicate scores in individuals in males (n = 12) and females in proestrus (n = 5), estrus (n = 6), and diestrus (n = 4) phase, respectively. (B) The mean immobility in males and females in each estrus phase were indicated by black triangles, dark gray circles, medium gray squares, and light gray diamonds, respectively. (C) Scores in individuals in males (n = 12) and females in proestrus (n = 4), estrus (n = 4) and diestrus (n = 7) were indicated by the same maker types with (A). (D) The mean immobility in males and females in each estrus phase were indicated by the same marker types with (B), respectively. Each asterisk indicates significant increasing the immobility from day 1 to 2. [file 12993_2017_126_MOESM4_ESM.tif]
